# The association of APOE with Alzheimer’s Disease in four Hispanic cohorts

**DOI:** 10.1002/alz.089512

**Published:** 2025-01-03

**Authors:** Giuseppe Tosto, Robert Barber, Nicole Phillips, Sid E. O'Bryant

**Affiliations:** ^1^ Columbia University, New York, NY USA; ^2^ University of North Texas Health Science Center, Fort Worth, TX USA

## Abstract

**Background:**

The long‐term goal of Health & Aging Brain Study – Health Disparities (HABS‐HD) is to establish population‐specific informed precision medicine for novel treatment and prevention strategies as has been done in other fields. Genomic studies are integral to these efforts and contribute vital data regarding genetic ancestry of the HABS‐HD participants, as well as whole genome sequence data, genome‐wide genotype (Illumina Global Screening array version 3.0) and epigenetic data (Illumina EPIC DNA methylation array).

**Method:**

We evaluated the association of *APOE* with Alzheimer’s Disease and Related Dementia (ADRD) in four different Hispanic populations: Mexicans in the Mexican Health and Aging Study (MHAS), Caribbean Hispanics in the Washington Heights Inwood Aging project (WHICAP) and Estudio Familiar de Influencia Genetica en Alzheimer (EFIGA), Peruvians in the Genetics of Alzheimer’s disease In Peruvian Populations (GAPP) and Mexican Americans from The Health & Aging Brain among Latino Elders (HABLE) and the Texas Alzheimer’s Research and Care Consortium (TARCC). Analyses were adjusted for age (≥60 years old), sex, and education. Global ancestry proportions, Native Americans (NAA), European (EUR), African (AFR) were estimated with ADMIXTURE software using the Human Genome Diversity Project as the reference panel. Specific secondary analyses of genetic ancestry were conducted using the same models.

**Result:**

The genetic ancestral composition was significantly different across cohorts. Peruvians and Mexicans showed a predominant NAA ancestry, Caribbean‐Hispanics appeared to have a major contribution of European and African ancestry, and Mexican‐Americans exhibited similar proportions of both European and Native ancestries. We observed a significant association with the ε4 allele dosage in all the cohorts. The strongest association between *APOE‐ε4* and risk for ADRD was observed in the Peruvian population (OR = 6.05, 95%CI = 3.01‐12.40). The genetic effect of at least one copy of the ε4 allele on ADRD risk was very similar in WHICAP+EFIGA, HABLE/TARCC and MHAS (OR = 2.52, 95%CI = 2.22‐2.85, OR = 2.86, 95%CI = 1.94‐4.22, OR = 2.76, 95%CI = 1.52‐5.02, respectively). Overall, random‐effect metanalysis estimated the *ε4* OR for ADRD at 2.56 (95%CI = 2.26‐2.87).

**Conclusion:**

To our knowledge, this is the largest collection of Hispanic participants phenotyped for ADRD. Our results showed a differential risk for ADRD from the *APOE‐ε4* allele across populations with admixed genetic ancestry.

